# Lipotoxicty in yeast: a focus on plasma membrane signalling and membrane contact sites

**DOI:** 10.1093/femsyr/foy034

**Published:** 2018-03-24

**Authors:** Patrick Rockenfeller, Campbell W Gourlay

**Affiliations:** 1 Kent Fungal Group, School of Biosciences, University of Kent, Canterbury, CT2 7NJ Kent, UK; 2 Institute of Molecular Biosciences, NAWI Graz, University of Graz, Humboldtstr. 50, 8010 Graz, Austria

**Keywords:** lipotoxicity, cell death, necrosis, apoptosis, necroptosis, Rim101, MAPK, diacylglycerol, ceramide, membrane contact sites

## Abstract

Lipotoxicity is a pathophysiological process triggered by lipid overload. In metazoans, lipotoxicity is characterised by the ectopic deposition of lipids on organs other than adipose tissue. This leads to organ dysfunction, cell death, and is intimately linked to lipid-associated diseases such as cardiac dysfunction, atherosclerosis, stroke, hepatosteatosis, cancer and the metabolic syndrome. The molecules involved in eliciting lipotoxicity include FAs and their acyl-CoA derivatives, triacylglycerol (TG), diacylglycerol (DG), ceramides, acyl-carnitines and phospholipids. However, the cellular transport of toxic lipids through membrane contact sites (MCS) and vesicular mechanisms as well as lipid metabolism that progress lipotoxicity to the onset of disease are not entirely understood. Yeast has proven a useful model organism to study the molecular mechanisms of lipotoxicity. Recently, the Rim101 pathway, which senses alkaline pH and the lipid status at the plasmamembrane, has been connected to lipotoxicity. In this review article, we summarise recent research advances on the Rim101 pathway and MCS in the context of lipotoxicity in yeast and present a perspective for future research directions.

## Teaser

In this review article, we summarise recent research advances on the topic of lipotoxicity in yeast and present a perspective for future research directions.

## INTRODUCTION

The adverse effects of excessive nutrition are a global socioeconomic and health-threatening burden of pandemic proportions. According to the World Health Organization (WHO), 39% of the adult world population (more than 1.9 billion adults) were overweight and 13% obese in 2016 (World Health Organization (WHO) 2018). Obesity is frequently associated with metabolic alterations that predispose individuals to co-morbidities such as insulin resistance, non-alcoholic fatty liver disease, and cardiovascular disease, together often referred to as metabolic syndrome (Unger *et al.*^2010^). A common pathological manifestation of metabolic syndrome is ectopic lipid accumulation in non-adipose tissues such as liver, heart, skeletal muscle and pancreas likely reflecting an oversupply and/or impaired disposal of fatty acids (FA) in these tissues. Although the exact aetiology of metabolic syndrome is not known, unbalanced lipid metabolism in non-adipose tissues is considered to contribute to lipotoxicity. The term lipotoxicity was first introduced in 1994 by Roger Unger to describe FA-induced β-cell degeneration in the context of non-insulin-dependent diabetes mellitus (Lee *et al.*^1994^). Lipotoxicity is now generally used to describe the process during which excess lipid accumulation in non-adipose cells and tissues results in cellular dysfunction, which may manifest as impaired cellular signalling, cellular stress responses and ultimately cell death. Multiple lipid species have been implicated in lipotoxicity including FAs and their acyl-CoA derivatives, TG, DG, ceramides, acyl-carnitines and phospholipids (Unger *et al.*^2010^).

The toxicity of these lipid species varies between cell types and depends on sub-molecular properties such as FA chain length and saturation. Exposure to saturated FAs (SFAs) such as palmitic acid and stearic acid triggers cell death at lower concentration than its unsaturated counterparts in mammalian cell culture models (Listenberger, Ory and Schaffer 2001; Listenberger *et al.*^2003^; Malhi *et al.*^2007^; Khan *et al.*^2012^). Often concomitant provision of monounsaturated FAs (MUFAs) has been shown to prevent the adverse effects of SFAs (Listenberger *et al.*^2003^). Poorer incorporation of SFAs versus MUFAs into TG may explain this phenomenon (Listenberger *et al.*^2003^). This means that MUFAs are not as accessible to the most effective detoxification route that is to withdraw lipids from phospholipid or ceramide synthesis pathways by conversion into TG and storage in lipid droplets.

Research in yeast has provided valuable insights into the toxicity associated with exposure to excess FAs, DGs, sterols and ceramides (Garbarino *et al.*^2009^; Petschnigg *et al.*^2009^; Rockenfeller *et al.*^2010^, ^2017^; Carmona-Gutierrez *et al.*^2011^; Fakas *et al.*^2011^). We will only briefly summarise knowledge on these lipotoxic triggers in the following paragraph, as this content has been extensively reviewed (Eisenberg and Büttner 2014).

FA oversupply in a *dga1*, *lro1*, *are1* and *are2* quadruple knock out (QKO) yeast strain, which is deficient for all DG and steryl acyl transferase activity triggers ROS production and cell death (Garbarino *et al.*^2009^; Petschnigg *et al.*^2009^; Rockenfeller *et al.*^2010^). In this situation, a cell would normally direct the excess FAs via the ER towards neutral fat (TG and SE) synthesis and storage. However, in the case of the QKO this is not possible, leading to the ER channelling surplus FA into phospholipid metabolism (Petschnigg *et al.*^2009^). This scenario generates massive ER-membrane stacks (Petschnigg *et al.*^2009^), ROS production (Rockenfeller *et al.*^2010^) and induces ER stress (Garbarino *et al.*^2009^). Mitochondria have been suggested as a source of ROS (Rockenfeller *et al.*^2010^) potential involvement of the ER-resident ROS-producing NADPH oxidase Yno1 (Rinnerthaler *et al.*^2012^: 1; Leadsham *et al.*^2013^: 1) still needs to be tested in this setting.

The toxic effects of excess FA exposure have been conflicting, with some pointing to either an apoptotic (Garbarino *et al.*^2009^) or a necrotic mode of death (Rockenfeller *et al.*^2010^). Ceramide and DG-induced cell death in yeast have been classified as necrotic (Carmona-Gutierrez *et al.*^2011^; Rockenfeller *et al.*^2018^). The current model of ceramide toxicity is mostly derived from *in vitro* models and involves ceramide-induced pore formation in the outer mitochondrial membrane (Birbes *et al.*^2001^; Siskind, Kolesnick and Colombini 2002, 2006; Samanta *et al.*^2011^). These ceramide pores may allow for unspecific protein release from the inter-mitochondrial space and thus facilitate the intrinsic apoptosis pathway (Siskind, Kolesnick and Colombini 2006).

In the following paragraphs, we attempt to provide a comprehensive picture of current lipotoxicity research in yeast focusing on DG as a lipotoxic trigger, Rim101 signalling as an important lipotoxic sensing and signalling pathway and the engagement of membrane contact sites (MCS).

## CONNECTING LIPOTOXICITY WITH CELL SIGNALLING, STRESS RESPONSE AND CELL DEATH

When cells die as a consequence of lipid overload, this can happen either accidentally or in a regulated fashion that is orchestrated by specific cell death pathways. The first case results in classical unregulated necrosis (accidental cell death), where cells undergo an uncontrolled functional decline that ultimately leads to the loss of plasma membrane (PM) integrity and thus the leakage of cellular content into the culture medium. In the second case, cells regulate their own demise in a properly organised fashion (regulated cell death). This modality includes yeast regulated cell death, regulated necrosis, programmed cell death, yeast apoptosis and autophagy-dependant cell death depending on the morphological features of cell death (Carmona-Gutierrez *et al.*^2010^, ^2018^; Eisenberg *et al.*^2010^). Even though these types of cell death can be differentiated, a full understanding of the molecular mechanisms regulating cell death is incomplete. Recently new routes of lipid-induced cell death have been revealed in yeast that could help us understanding basic cell death regulatory mechanisms involving lipids such as ceramide, DG or FA and indeed lipotoxicity itself.

## SENSING LIPID STRESS AT THE PM

It is important to understand how yeast cells sense lipid stress and elicit an appropriate response. *S. cerevisiae* offers a number of adaptive response stress pathways that respond to lipid stress, most notably the mitogen-activated protein kinase (MAPK), yeast protein kinase (Ypk1/2) and Rim101 pathways. These pathways sense changes at the PM and transduce the signal into an intracellular response. The five MAPK pathways regulate the pheromone response, filamentation/invasion, high osmolarity growth, spore wall assembly and cell wall integrity (CWI) (Chen and Thorner 2007). From these five pathways, CWI signalling has highest relevance for lipotoxicity. Lipids can interfere with this pathway either on the extracellular through changes in PM tension and activation of stretch receptors or through the manipulation of phosphatidylinositol-4,5-bisphosphate (PI4,5P_2_) distribution or recruitment and/or activation of Pkc1 (Levin 2011). CWI signalling is generally known as an adaptive response pathway and is thus rather considered as a protective mechanism; however, interference with this pathway has been connected to cell death induction (Lommel, Bagnat and Strahl 2004; Badrane, Nguyen and Clancy 2016).

An alternative pathway activated by PM-stress is mediated by yeast protein kinases (Ypk1/2) downstream of TOR complex 2. Interestingly this mechanism involves Lem3-dependent lipid remodelling (which will be explained in more detail in the Rim101 paragraph below) to establish Rho1 recruitment to the PM. As part of the CWI pathway (see above), Rho1 is recruited to the PM by PI4,5P_2_. Hence, the Ypk1/2 pathway can be regarded as a backup signalling mechanism for PI4,5P_2_-free Rho1 recruitment to cover for stress-induced loss of PI4,5P_2_ (Hatakeyama, Kono and Yoshida 2017). Ypk1 has further been shown to regulate FA uptake and energy homeostasis through regulating endocytosis (Jacquier and Schneiter 2010).

### The Rim101 pathway

The Rim101 pathway was initially introduced as a fungal adaptive response to alkaline pH (Futai *et al.*^1999^; Maeda 2012; Serra-Cardona, Canadell and Ariño 2015). The last decade of research on this topic has revealed that the Rim101 pathway is not limited to regulating the alkaline pH response but that it can also sense lipid stress. As such, it can detect lipid alterations at the PM or rearrangements of the asymmetrical lipid distribution among the two leaflets of the bilayer (Ikeda *et al.*^2008^; Obara, Yamamoto and Kihara 2012). The outer leaflets of all eukaryotic PMs are enriched in complex sphingolipids and phosphatidylcholine (PC), whereas phosphatidylethanolamine (PE), phosphatidylserine (PS), phosphatidylinositol (PI) and phosphatidic acid (PA) are the predominant building blocks shaping the inner leaflet (Devaux 1991; Verkleij and Post 2000). The uneven distribution of zwitterionic (PC, PE) and anionic lipids (PS, PA) within the two PM leaflets thus establishes lipid asymmetry and creates a non-zero potential difference between the two sides of the PM (Gurtovenko and Vattulainen 2008). Lipid asymmetry is important for cellular processes such as vesicular transport (Chen *et al.*^1999^), cytokinesis (Emoto and Umeda 2000) and removal of apoptotic cells (Fadok *et al.*^1992^). The PM lipid asymmetry is actively achieved and maintained by the activity of ATP-dependant flippases and floppases, which represent enzymes facilitating flip (outward-directed) or flop (inward-directed) translocation reactions of lipids within a membrane (Pomorski *et al.*^2003^; Ikeda, Kihara and Igarashi 2006).

**Figure 1.**
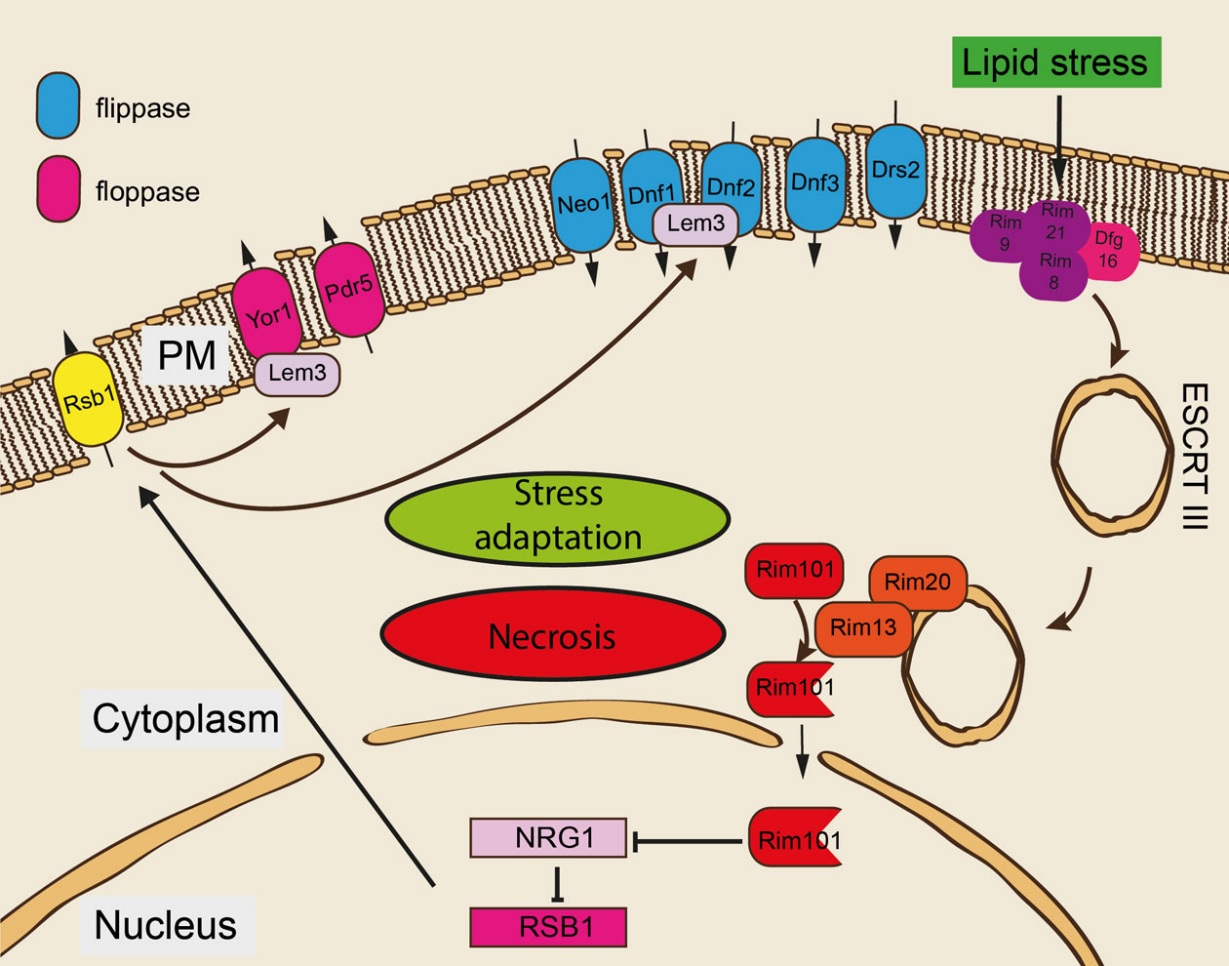
The Rim101 pathway mediates a response to lipid stress: adaptation or necrosis. Lipid stress is sensed by the Rim21-sensing complex consisting of Rim8, Rim9, Rim 21 and Dfg16. The sensing complex triggers activation of the Rim101 pathway which depends on ESCRT III. The cysteine-protease Rim13 cleaves the carboxy-terminus of Rim101 and thus activates it as a transcriptional repressor. Activated Rim101 regulates gene expression of *NRG1* and *RSB1* among others. *RSB1* itself encodes a sphingoid long chain base transporter. Rsb1 also regulates PM-flippase (Yor1) and floppase activities (Dnf1 and Dnf2) via Lem3. This may facilitate adaption to PM-lipid stress or trigger necrotic cell death.

The Rim101 pathway contains a sophisticated sensor complex that can sense lipid alterations at the PM. The sensor complex consists of Rim8 and the three transmembrane proteins Dfg16, Rim9 and Rim21. The carboxy-terminal cytosolic domain of Rim21 localises to the PM under normal conditions, whereas the lipid perturbation of the PM triggers its dissociation (Nishino, Obara and Kihara 2015). The activation of the sensor complex induces a proteolytical complex, which consists of Rim13, Rim20, Ygr122w and Rim101 (Maeda 2012). Rim13 proteolytically cleaves and thereby activates the transcriptional repressor Rim101 (Futai *et al.*^1999^). The activation of the Rim101 pathway by lipid stress has been investigated in a number of studies including (i) genetic deletion of flippases and floppases or their upstream regulators (Ikeda *et al.*^2008^; Obara and Kihara 2014; Nishino, Obara and Kihara 2015) (ii) addition of palmitoleic acid (Richard *et al.*^2014^) (iii) expression of phospholipase A2 (Mattiazzi *et al.*^2010^) and most recently by (iv) excess DG (Rockenfeller *et al.*^2018^). Whether the adverse effects of lipid overload in the QKO or similar models also depend on Rim101 signalling and whether PM asymmetry is disturbed still remains to be tested. Also, the specific effects of lipotoxic triggers on PM asymmetry are not very well documented in the literature. In most of the above-cited studies, it appears that Rim21 senses changes in the PM's lipid asymmetry in a similar way as under alkaline conditions. This is not surprising as both conditions, alkaline and lipid stress, can actually interfere with the charge gradient at the PM. Disturbance of specific physicochemical properties of the PM can be perceived by the sensing complex involving Rim21 under both conditions of stress. This sensing mechanism allows the cell to adapt to a changing environment—be it a change in ambient pH or the lipid environment. Whether lipotoxic triggers such as DG or palmitoleic acid trigger the Rim101 pathway directly at the PM or first enter the cell and then trigger the pathway from within remains unclear. The latter option has recently been introduced by Obara and Kihara. They found that Rim101 signalling can be triggered by ER stress (Obara and Kihara 2017). ER stress can affect lipid asymmetry and thus Rim101 could be triggered in order to compensate for these changes. Once the Rim101 pathway is activated by the Rim21 sensing complex, it triggers carboxy-terminal cleavage of Rim101 by Rim13, which uncovers its gene repressing activity (Fig. 1). Rim101 processing is further dependent on Snf7, which is a component of the endosomal sorting complex required for transport III (ESCRT III). Rim20 is thought to function as an adaptor, bringing Snf7 into close proximity of Rim13 to facilitate Rim101 cleavage. A number of Rim101-repressed genes have been identified (Lamb and Mitchell 2003) among which *NRG1* connects to functional lipid changes at the PM. It is likely that the activation of the Rim101 pathway in response to lipid stress facilitates changes in lipid composition at the PM to facilitate adaptation to the environment. For example, the rearrangement of PM lipids can be achieved via altered expression or activation of PM lipid translocases such as Rsb1, Yor1, Dnf1 and Dnf2 (Kihara and Igarashi 2004). However, the abrogation of the Rim101 pathway has been shown to prevent cell death in a number of lipotoxic settings (Richard *et al.*^2014^; Rockenfeller *et al.*^2018^), which at first glance seems counterintuitive. A potential explanation for this could be that active Rim101 signalling is needed to efficiently direct lipids from the PM to the ER. If the pathway is abrogated, lipids might not be delivered and hence toxic effects downstream of Rim13 and Rim101 can be prevented. This hypothesis would suggest that the Rim101 pathway may act not only as a detector of PM lipid stress, but also as a regulator of lipid transport. In addition, in the case of DG and palmitoleic acid stress, Rim101 activity may also actively disrupt PM integrity to promote necrosis (Richard *et al.*^2014^; Rockenfeller *et al.*^2018^). These findings raise the possibility that yeast cells may initiate a Rim101-dependent form of necrosis if cells are unable to adapt to lipotoxic conditions.

## THE ROLE OF MCS IN LIPOTOXICITY

**Figure 2.**
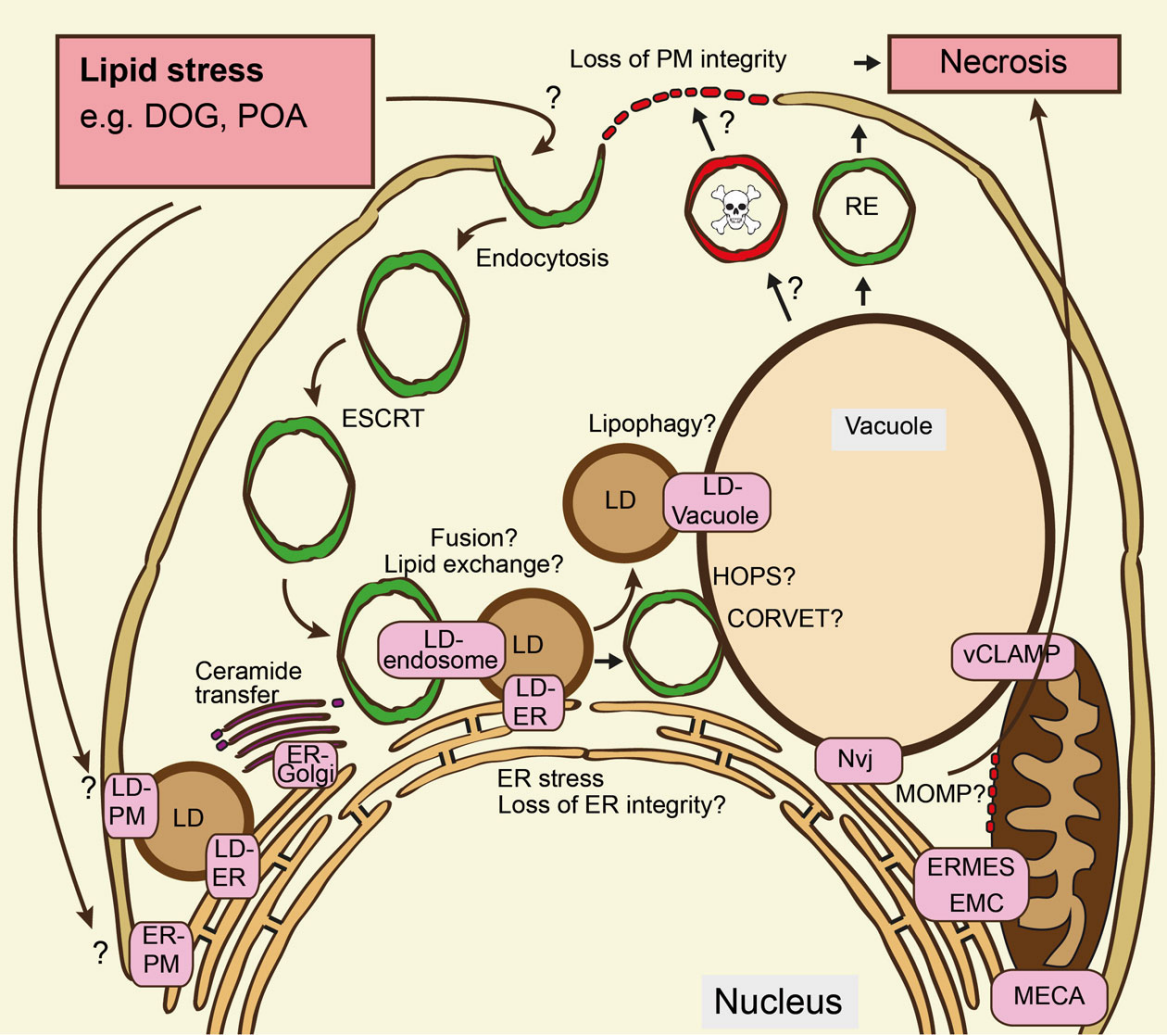
Perspectives view of how lipid traffic could affect cellular lipotoxicity. Externally supplied lipids such as palmitoleic acid (POA) and dioctanoyl glycerol (DOG) trigger necrosis in yeast. Here, we schematically depict the potential cellular lipid trafficking routes and potential involvement of vesicular and MCS-mediated transport. Lipids can be internalised by endocytosis, direct interaction with PM-lipids or receptor/translocase-mediated (not shown) pathways. Once internalised, lipids may be transported via the vesicular ESCRT-dependent trafficking route or via contact sites. Lipid-containing endosomes can interact and exchange lipids with the ER and/or LD, which could further deliver lipids to other organelles including the vacuole, mitochondria and Golgi via fusion events or lipid exchange through MCS. Lipids can trigger ER stress and potentially disrupt ER-membrane integrity. In order to alleviate from ER-stress/toxicity, toxic lipids could be channelled into the Golgi, mitochondria and LDs for metabolisation or storage purposes. LDs can remobilise stored lipids via lipolysis or lipophagy. Delivery of external-lipid-containing-endosomes can fuse with the vacuole possibly involving HOPS/CORVET or other SNARE/Rab machineries. Toxic lipids could then be delivered to the PM via recycling endosomes and thus disrupt PM integrity. Toxic lipids reaching the mitochondria could induce MOMP and thus induce cell death via mitochondrial pathways.

Research over the last decade has shown that lipid transport is not limited to vesicular and protein-mediated transport, but also involves non-vesicular inter-organelle lipid transfer via MCS (Prinz 2014; Gatta and Levine 2017). It has become apparent that virtually all cell organelles are interconnected in a dynamic MCS-network, which effectively participates in signalling and metabolic channelling of substrates between organelles (Prinz 2014; Quon and Beh 2016) (Fig. 2). The MCS that have been described in yeast so far include the ER mitochondria encountering structure (ERMES) (Kornmann *et al.*^2009^) and ER-mitochondria contact (EMC) (Lahiri *et al.*^2014^), mitochondrial inner with outer membrane (Harner *et al.*^2011^), vacuole and mitochondria patch (Hönscher *et al.*^2014^), ER-PM contact site (Stefan *et al.*^2011^; Manford *et al.*^2012^), nucleus vacuolar junction (NVJ) (Pan *et al.*^2000^; Toulmay and Prinz 2012), ER-GOLGI contacts (Liu *et al.*^2017^), mitochondria-ER-cortex anchor (MECA) (Lackner *et al.*^2013^) and ER-peroxisome contacts (Munck *et al.*^2009^). MCS are characterised by the presence of so-called tethers, which are proteins or protein complexes that simultaneously bind the membranes of two distinct organelles to bring them into close proximity and physically tether them. Excellent overviews of MCS-tethering proteins are already available (Prinz 2014; Gatta and Levine 2017). In the following paragraphs, we will focus on a small selection of MCS, which provide considerable relevance for lipotoxicity.

### Nvj2 facilitates ceramide transfer at ER-Golgi contact sites

MCS are of particular interest in lipotoxicity research as they may give key answers as to how toxic lipids are transported, metabolised and distributed within the cell and how lipids can participate in cross-organelle signalling. A recent study revealed that Nvj2 tethers the ER and medial-Golgi (Liu *et al.*^2017^). Nvj2 localises to the ER and is enriched at the nucleus-vacuole junction under unstressed conditions. However, during ER stress Nvj2 disappears from the NVJ and induces novel contacts between the ER and medial-Golgi. The authors of the study suggest that the establishment of such contacts could meet the purpose of effectively channelling toxic ceramide, which is known to increase upon ER stress, from the ER to the Golgi. This would represent an ER-protective mechanism to prevent from toxic ceramide accumulation within the ER (Liu *et al.*^2017^). Interestingly, Nvj2 contains a synaptotagmin-like mitochondrial lipid-binding protein (SMP) domain (Toulmay and Prinz 2012), which could allow for lipid transfer between membranes. Such activities have been demonstrated by SMP domain containing proteins before (Schauder *et al.*^2014^; AhYoung *et al.*^2015^; Saheki *et al.*^2016^; Yu *et al.*^2016^). The exact mechanism of how ceramide facilitates cell death is still illusive but there is some good evidence that C16-ceramide can trigger pore formation in the outer mitochondrial membrane (Siskind and Colombini 2000; Siskind, Kolesnick and Colombini 2006). This mitochondrial outer membrane permeabilisation (MOMP) could allow for the release of apoptogenic or necrosogenic cell death signals from mitochondria (Siskind, Kolesnick and Colombini 2002) such as cytochrome C (Manon, Chaudhuri and Guérin 1997; Ludovico *et al.*^2002^), apoptosis inducing factor (Wissing *et al.*^2004^) and endonuclease G (Büttner *et al.*^2007^) or simply lead to ATP depletion and/or dysfunctional phospholipid supply to the PM culminating in necrosis. Future research should address the questions whether ERMES or EMC contact sites are essential for ceramide-induced MOMP *in vivo* and whether ceramide accumulation in the ER also induces pore formation in the ER or rather transmits its adverse effects via the mitochondrial route. To protect from ceramide accumulation in the ER, ceramide can be converted into acylceramides by the acyltransferases Dga1 and Lro1 (Voynova *et al.*^2012^). Acylceramides are then stored in LDs. Accordingly, the deletion of *DGA1* and *LRO1* increases ceramide accumulation in the ER upon ER stress in the *NVJ2* deletion mutant that increases toxicity. However, the detoxification pathways via Dga1/Lro1 or Nvj2 are redundant as only the triple deletion mutant shows significant ceramide increase (Liu *et al.*^2017^). Lipid detoxification via Dga1 and Lro1 is not limited to ceramide, but also extends to DG (Rockenfeller *et al.*^2018^) and FA (Garbarino *et al.*^2009^; Petschnigg *et al.*^2009^; Rockenfeller *et al.*^2010^). Whether Nvj2 can also facilitate DG transfer or ameliorate FA stress remains to be tested.

### ER-PM contact sites

ER-PM contact sites represent MCS where the ER comes into close proximity to the PM. The average distance of the two contacting membranes is about 33 nm (Pichler *et al.*^2001^; West *et al.*^2011^) and tethering is achieved through at least six different proteins, namely Tcb1, Tcb2, Tcb3, Scs2, Scs22 and Ist2 (Manford *et al.*^2012^). A yeast mutant deleted in the six genes encoding these tethers (Δ-tether mutant) is disrupted in ER-PM contacts and is thus a valuable tool to study ER-PM-dependent lipid transfer, which has been described to occur at these sites (Stefan, Manford and Emr 2013). Sterol transport that is facilitated by a conserved family of oxysterol-binding (OSBP)-related proteins (ORPs) has been thoroughly studied at these sites (Lev 2010; Toulmay and Prinz 2011). ORP proteins contain a conserved sterol-binding domain as well as specific domains that regulate targeting to ER–PM contacts such as pleckstrin homology domain that bind PI_4_P in the PM (Roy and Levine 2004) and the FFAT (two phenylalanine residues in an acidic tract) motif that interacts with vesicle associated membrane protein-associated protein in the ER membrane (Loewen and Levine 2005).

A recent study investigated whether Rim101 signalling depends on ER-PM contact sites (Obara and Kihara 2017). The authors revealed that Rim101 signalling occurred mostly at sites different from ER–PM contact sites. However, Rim101 signalling was constitutively activated in the Δ-tether mutant. Since loss of ER–PM contact sites has been shown to induce ER stress (Manford *et al.*^2012^), the authors also tested whether the Rim101 pathway was activated as a consequence of ER stress. Indeed the conducted experiments confirmed that Rim101 signalling is triggered in order to facilitate adaptation to ER stress at the level of PM lipid organisation.

### LD contact sites and their potential involvement in lipotoxic signalling

In addition to the above-listed contact sites, LDs can also engage in similar cellular substructures that are summarised in a recent review (Schuldiner and Bohnert 2017). These LD-contact sites include interactions with organelles such as the ER (Markgraf *et al.*^2014^; Mishra *et al.*^2016^; Wang *et al.*^2016^), mitochondria (Wang *et al.*^2011^), peroxisomes (Binns *et al.*^2006^), endosomes (Guimaraes *et al.*^2015^), vacuole (Wang, Miao and Chang 2014a; Barbosa *et al.*^2015^), inclusion bodies (Moldavski *et al.*^2015^) or homotypic interactions with a second LD (Binns *et al.*^2006^; Wang, Miao and Chang 2014b). However, these contacts are slightly different to conventional MCS due to the nature that LDs do not contain a double-leaflet-membrane with an aqueous core but only a single layer of phospholipids covering the neutral lipid core (Wang 2015). LDs originate from the ER; hence, the existence of an LD-ER contact is rather obvious. It became apparent that not only in yeast cells nearly all LDs remain connected to the ER since even mature LDs are accessible to luminal ER proteins (Mishra *et al.*^2016^). An additional special feature of LD-ER contacts is the prevalence of lipidic bridges, which represent continuous phospholipid monolayer surrounding the LD and outer leaflet of the ER. These lipidic bridges do not seem to be sufficient to structurally maintain contacts, which suggest the existence of additional proteinaceous tethers (Schuldiner and Bohnert 2017). Even though particular tethering complexes at LD contact sites have not been clearly identified so far, some proteins, which are potentially part of such complexes, have been proposed to facilitate contacts (Schuldiner and Bohnert 2017). As such Seipin (Sei1/Fld1) is potentially involved in stabilising LD-ER contacts (Wang, Miao and Chang 2014b; Grippa *et al.*^2015^) and the ER acyl-CoA synthetase FATP1 and the LD-resident diacylglycerol acyltransferase (DGAT2) have been proposed as ER-LD tethers in *C. elegans* (Xu *et al.*^2012^). The ER-resident protein Ice2 has further been identified as an important regulator of lipid metabolism at LD-ER contact sites (Markgraf *et al.*^2014^). Ice2 has a cytosolic domain with affinity to lipid LDs. It facilitates TAG mobilisation in early exponential growth phase and TAG synthesis during early stationary phase via transfer of Dga1 from ER to LDs. Controlling LD's access to Dga1, which represents the major DG-acyltransferase, Ice2 determines TAG synthesis and LD-size.

At least some of these LD contact sites might be of importance for cell death signalling under lipotoxic conditions. LDs are to be understood as buffers to cope with lipid overload. As such the connection to the ER is of major importance to facilitate alleviation from lipid stress within the ER (Liu *et al.*^2017^). However, LDs might also serve as a source to deliver toxic lipids to trigger regulated cell death. For instance, the connection between LDs and mitochondria allows for FA delivery to the mitochondrion. This MCS could possibly further facilitate MOMP via supply of ceramide to establish pore formation as described above. This could lead to apoptotic or necrotic outcomes. LD-PM contact sites might be actively involved in lipid supply to the PM bilayer possibly joining forces with the ER. Lipid stress could potentially affect the function of LD-PM-contacts and thus inhibit lipid transport across contact sites. This could disrupt the dynamics of PM lipid homeostasis and further trigger loss of PM integrity in necrotic scenarios. These potential involvements of LD contact sites in the regulation of cell death are ready to be addressed in future research questions and will doubtless increase our understanding of lipotoxicity and cell death. An interesting question is for example whether Seipin-regulated PA metabolism (Wolinski *et al.*^2015^) is required in diverse settings of lipotoxicity such as DG or palmitoleic acid stress.

## CONCLUSION AND OUTLOOK

The Rim101 pathway is established as an important lipid responsive pathway. However, important questions need to be addressed in future research: Can Rsb1/Lem3-dependent translocase regulation account for the full effects of Rim101-dependent cell death or are there other downstream effectors of the Rim101 pathway that are responsible for cell death under conditions of lipid stress? Transcriptional control by the Rim101 pathway has been proposed to account for the downstream adaptations at the PM in response to lipid stress (Ikeda *et al.*^2008^; Richard *et al.*^2014^). However, the transcriptional control of Rim101 signalling might only reflect one side of the coin. We are currently investigating whether additional events such as lipid transport, metabolism and storage succumb to lipotoxic cell death and whether these are situated up- or downstream of Rim101 signalling. It may be the case that the generation of lipotoxicity is dependent upon the sum of transcriptional responses, lipid metabolism, trafficking and presence of cell stress (Fig. 2). In response to this, a number of unanswered questions are revealed: how does vesicular lipid traffic interact with contact site-mediated lipid transfer?; what are the dynamic changes of contact sites as a response to lipid stress and how does that affect cell fate? Answers to questions such as these will be a key to understanding lipid metabolism and homeostasis in its entity and thus lipotoxicity itself. Such advances will not only offer new perspectives for drug development, but also add to our general understanding of lipid and membrane biology in healthy and disease states.
